# Deploying a Wireless Sensor Network to Track Pesticide Pollution in Kiu Wetland Wells: A Field Study

**DOI:** 10.3390/s25134149

**Published:** 2025-07-03

**Authors:** Titus Mutunga, Sinan Sinanovic, Funmilayo B. Offiong, Colin Harrison

**Affiliations:** School of Engineering and Built Environment, Department of Electrical and Electronics, Glasgow Caledonian University, Cowcaddens Road, Glasgow G4 0BA, Scotland, UK; sinan.sinanovic@gcu.ac.uk (S.S.); funmilayo.offiong@gcu.ac.uk (F.B.O.); colin.harrison@gcu.ac.uk (C.H.)

**Keywords:** wireless sensor network, LoRaWAN, signal propagation, pesticide, water, wireless, sensors, pollution, contamination

## Abstract

Water pollution from pesticides is a major concern for regulatory agencies worldwide due to expensive detecting mechanisms, delays in the processing of results, and the complexity of the chemical analysis. However, the deployment of monitoring systems utilising the internet of things (IoT) and machine-to-machine communication technologies (M2M) holds promise in overcoming this major global challenge. In this current research, an IoT-based wireless sensor network (WSN) is successfully deployed in rural Kenya at the Kiu watershed, providing in situ pesticide detections and a real-time data visualisation of shallow wells. Kiu is an off-grid community located in an area of intensive agriculture, where residents face a high exposure to pesticides due to farming activities and a reliance on shallow wells for domestic water. The evaluation of path loss models utilising channel characteristics obtained from this study indicate a marked departure from the continuous signal decay with distance. Transmitted packets from deployed sensor nodes indicate minimal mutations of payloads, underscoring systems reliability and data transmission integrity. Additionally, the proposed design significantly reduces the time taken to deliver pesticide measurement results to relevant stakeholders. For the entire monitoring period, pesticide residues were not detected in the selected wells, an outcome validated with lab procedures. These results are attributed to prevailing dry weather conditions which limited the leaching of pesticides to lower layers reaching the water table.

## 1. Introduction

The advancement in sensor technology and improved networking capabilities have spurred research in IoT water quality solutions [1,2,3]. Sensors play an integral role in detecting environmental parameters by generating signals in response to changes in the state of their embedded elements. Impressive gains were observed by the development of wireless sensors, making it possible to deploy them with a minimal cost of infrastructure and maintenance due to a lack of physical connections. Notably, wireless sensors can be centrally accessed, improving the management of monitoring systems [4]. Additionally, advances in communication technologies by the acquisition of rapid data transmission rates, greater network support, and increased power efficiency have seen their wide acceptance in remote monitoring scenarios. However, despite this remarkable achievement in the realm of environmental monitoring, gaps still exist, particularly in monitoring pesticide pollution. Pesticides are a class of compounds that are mostly organic in nature and do not induce any noticeable physicochemical changes targeted for detection by available off-the-shelf sensors [5]. These chemicals are deliberately introduced in the environment for protection against crop pests and diseases. They are particularly harmful since they are designed to eliminate plant and animal life and when used indiscriminately can pose a significant risk in biodiversity. Pesticides and fertilisers are a common cause of water pollution in rural areas as a result of agricultural runoff [6]. They contaminate water, an essential commodity for everyday activities such as hygiene, cooking, and sanitation. Beyond anthropogenic uses, water plays a vital role in maintaining aquatic ecosystems, which not only serve as habitats for biotic communities but also provide ecological services such as climate stabilization, flood control, and water purification [7]. Due to its limited supply, caused partly by scarce water resources and the contamination from both natural and human activities, water regulatory bodies around the world have implemented the routine monitoring of their water quality to ensure compliance with health and safety standards [8]. For decades the surveillance of pesticide pollution in aquatic ecosystems has relied on grab sampling strategies, often producing data of a low spectral resolution despite the dynamic nature of water sources on sub-daily temporal scales [9,10]. This leads to missing short spikes of pollution events and a failure to identify fine-scale temporal variations.

Clinical features of the exposure to pesticide exhibit both acute and chronic effects. Immediate health effects include salivation, emesis, sweating, urination, paralysis, allergic reactions, ataxia, hypothermia, and even death in severe situations [11], while long-term exposure can lead to endocrine disruption, reproductive harm, birth defects, cancer, and neurological effects [12,13]. Pesticide residues migrate from treatment sites to other non-target areas, often causing harm to the environment. Weather conditions have a great impact too on the spread of pesticides into the environment [14]. Wind blows pesticide mists into new areas while precipitation promotes their leaching deeper into the ground, eventually reaching the water table [15].

Despite the well-documented effects of these chemicals, regulatory systems may fail to enforce strict controls on agricultural malpractices, allowing contamination to continue unchecked. A relevant example is in agricultural economies like Kenya, where the extensive use of pesticides in arable areas still raises major environmental and public health issues. Between 2015 and 2018, Kenya’s pesticide imports show a dramatic increase from 6400 to 15,600 tonnes, where 76% of them are regarded as Highly Hazardous Pesticides (HHPs) [16]. The country also faces other challenges of demographics and settlement which further complicates pesticide contamination. Over 70% of the country’s population reside in the rural areas where agriculture is practiced [17]. With subsistence farming commonly conducted by women and children, studies indicate that they have lower literacy skills than men; hence, they find it difficult to understand pesticide names and interpret safety information labels, increasing their vulnerability [18,19]. In addition, water continuous to be a scarce commodity in the rural areas since they lack piped water infrastructure. Over 41% of the rural population depend on shallow wells, springs, and ponds for their domestic water needs [20].

This paper introduces a low-cost, IoT-based pesticide monitoring solution for rural communities in Kenya. The current study focuses on the Kiu wetland, in Makueni County, a hub for horticultural activity as well as a water catchment area. The dual use of the wetland has made it prone to pesticide contamination and hence requires constant water quality monitoring. The existing monitoring frameworks fall short in handling the dynamic and widespread nature of the contamination in the watershed area. The Kiu community is an off-grid rural area where agriculture serves as a primary source of livelihood. This puts them in a vulnerable situation by exposing them to dangers of pesticide poisoning in a country where pesticide monitoring is not performed. Traditionally, nations evaluate water quality using laboratory-based analytical techniques—which entail sample collection and transportation to specialised facilities for chemical analysis. Although methods such as gas chromatography–mass spectrometry (GC-MS) and liquid chromatography–mass spectrometry (LC-MS) remain superior in terms of their low detection limits, great accuracy, and high specificity, they have main shortcomings [21,22,23]. Identified challenges include expensive equipment, labour-intensive operations, long turnaround times, and sophisticated instrumentation, making them unsuitable for rapid, in situ detections [24,25,26]. Moreover, these approaches rely on spot sampling, which frequently produces poor spatial and temporal densities in datasets unsuitable for trace pollutants. Extrapolating overall contamination trends from few sample sites runs the danger of obtaining false conclusions given the dispersed character of water sources in the research area. Thus, a surrogate approach is therefore required to address this issue under investigation.

Detecting pesticides in water calls for precise and quick response systems. Within the research community, the use of wireless sensor networks (WSNs) in environmental monitoring has attracted a lot of interest since it presents a promising data-collecting infrastructure [27]. The incorporation of the internet of things (IoT) in wireless sensing promotes data sharing possibilities, further enhancing data accessibility. An IoT-based system usually consists of many tiny sensor nodes spread across wide geographical areas connected to a base station using a networking protocol, with the base station linked to the internet for resources. Microcontrollers form part of the sensor nodes to control the sensing operations by executing programs stored in their internal memory. Base stations usually have a higher processing capability to enable them to analyse the data or act as a gateway to external servers for further computations and visualisations. IoT-based solutions are indeed low-cost, real-time, remote, scalable, and efficient in distant water quality monitoring [28]. However, as the number of sensors increase in a monitoring scenario, the service delivery is threatened. Advanced techniques like time switching nonorthogonal multiple access (TS-NOMA) can be applied to alleviate such bottlenecks [29].

Numerous research projects for water quality monitoring have been conducted utilising technologies such as Bluetooth [30], long range (LoRa) [31], Wi-Fi [32], the narrow-band internet of things (NB IoT) [33], and radio frequency identification (RFID) [34]. Choosing between these networks is determined by considering factors such as the energy needs, transmission speed, communication range, and deployment costs. Bluetooth technology supports the IEEE 802.15.1 standard. The devices use adaptive frequency hopping, a desirable feature for avoiding potential interference from microwaves or Wi-Fi by skipping occupied channels [35]. With a range of 200 m and 2 Mbps transmit speeds, the technology is not suitably adapted for long-range outdoor conditions typical in rural areas. The Wi-Fi standard 802.11a utilises orthogonal frequency-division multiplexing (OFDM) to enable high-throughput data rates ranging from 6 to 54 Mb/s with a range of up to 200 m while the newly released Wi-Fi 7 promises speeds of up to 30 Gbps. The devices however have high energy demands compared to Zigbee and Bluetooth, limiting their suitability as end nodes, but they are very useful for accessing the internet in monitoring scenarios [36]. Cellular technologies including the NB-IoT and LTE-M adapted for IoT ecosystems need the frequent synchronization of their devices, leading to high power demands [37]. Both Ingenu and Sigfox technologies, though designed to support IoT applications by optimising their range and battery efficiency, are not open standards and hence require subscriptions to use their network [38,39].

Modelling is an essential phase in the design and deployment of environmental sensors, as it provides insights into the field expectations beforehand [40]. The network designer can take advantage of the offered opportunity to optimise their system performance and refine their design before the actual deployment. Effective radio frequency planning requires a proper understanding of the spatial and temporal characteristics of signal propagation to facilitate a comprehensive network design and diagnostics. As a result, a myriad of path loss models have been developed to design, map, and optimise the signal performance across different environments. The accuracy of their analysis largely depends on the models’ capability to precisely predict the network coverage and signal strength. Several studies have modelled LoRa signal propagation using established models such as Okumura–Hata [41], COST 231-Hata [42], log-distance [43], log-normal [44], and Stanford University Interim (SUI) models [42]. Empirical models, despite their broad application due to their simplicity, have been reported to yield less accurate predictions of path loss compared to analytical approaches [45].

This current article presents the implementation of a wireless network that employs paper-based sensors to detect the presence of pesticides in shallow wells and surface water. The essential data for this project includes the type of pesticide, the time of detection, and the location. While this information is valuable for stakeholders, the equipment should also report the signal strength and traffic flow to support further analysis. Radio planning for the feasibility of the LoRa application in the area under investigation is also conducted. This approach addresses the challenge of monitoring a highly distributed network of water sources, necessitating a low-cost detection method. The data is captured by sensor nodes equipped with RFID readers and tags, each preloaded with the names of various pesticides. RFID technology, traditionally used for object recognition, enables the transmission of small data packets, making it suitable for this application. As dedicated plug-and-play sensors for pesticide detection are not commercially available [46], the sensor nodes are custom-designed, incorporating multiple communication technologies to meet the specific needs of the system. Data packets are sent to the server by the LoRa connection. In remote locations, where there is typically a lack of necessary infrastructure and poor network connectivity, LoRa technology proves to be a perfect solution for data transfer. Apart from other accessible technologies, LoRa provides a larger communication range, is affordable, and resists multipath fading [47].

## 2. Related Work

Reports from previous studies on water quality monitoring utilising LoRa technology have majorly focussed on elaborate schemes of data visualisation and strategies of data management schemes [48,49]. Liloja et al. [50], while monitoring two reservoirs for four water parameters including the temperature, turbidity, conductivity, and potential of hydrogen (pH), utilised the ThingSpeak cloud service for their analysis and graphical representation. Their approach enabled a seamless visualisation on web browsers and user smartphones. The group faced long queues and delays in transferring data to the cloud in real-time as the sensor nodes were configured to collect data in seconds and transmit in minutes. A similar study by Hsieh et al. [51], monitoring the same set of parameters in an ecological pond, utilised a Raspberry Pi to create a MySQL database facilitating the storage of data and its presentation into a website. However, their system was only applicable to areas with Wi-Fi network access, while ignoring underserved remote areas with scant communication infrastructure. In other research, as reported by Hang and colleagues [52], the monitoring of algal blooms and water quality incorporated a pre-processing stage to reorganise the multiparameter data collected from various sensors before transmission. Task assignments were assigned to the microprocessor, where ATmega328p handled sensing and the controlling of the sampler for water, while AT mega 3204 was reserved for formatting the data. The study measured parameters including the global positioning system (GPS), turbidity, pH, and water images captured by the cameras. Although the utilisation of two microcontrollers improved the workflows of the system, they led to an increase in the energy demand of the system. Swing effects were observed with the unmanned aerial vehicle (UAV) even when the speed was 1 m/s, raising concerns about possible risks of sample spills. Additionally, the movement of the mechanical parts for activating the sampler was uncertain, highlighting the need for a better mechanism. Aquez et al. [53] similarly included a repackaging strategy of the data before uploading to the message queuing telemetry transport (MQTT) broker. Their study also included two microcontroller units (MCUs), one dedicated to establishing the LoRa connection and another for the ethernet connection. In their study, efforts were dedicated to detecting anomalous data using the anomaly detection toolkit package. The visualisation of the collected data was facilitated using Amazon web services, where variations in water parameters were observed through Grafana. Coverage gaps were observed during radio planning, and repeater stations were included in the network. This, however, adds possible complexity in the data aggregation and transmissions by the repeater stations. In addition, these nodes are always in active listening mode for packets from other nodes, or complicated wake-up mechanisms need to be implemented to maintain the power consumption at typical levels.

Studies have indicated that nodes spent more energy while communicating than in sensing or processing phases [54,55]. To address this issue, Merin and colleagues [56] designed their system to dynamically adjust the power spent by nodes in transmission based on the distance from the gateway. In their approach, a GPS module attached to the gateway calculates the distance between itself and a mobile node. An algorithm is then implemented to reduce the transmit power of the nodes as they approach the gateway. This optimisation extends the lifetime of the battery and network too. They monitored parameters including the pH, turbidity, and temperature, which were then visualised using ThingSpeak. While the adaptive transmission power control algorithm achieved a 40% energy minimization margin, the value was in comparison to a static parameter setting of Tp = 17 dB and SF = 12; other parameter settings were not evaluated to establish their performance. Similarly, Baghel et al. [57], focussing on minimizing the energy consumption by sensor nodes, implemented a GPS sensor using an RSSI-based algorithm for localization. Their work involved monitoring the pH, temperature, TDS, and electrical conductivity in the IIT Roper campus and the Satluj River. To enhance the accuracy, they used a Kalman filter for RSSI values in Satluj to minimize the noise and improve the localization precision. 

Most studies investigated the physicochemical parameters to monitor water quality. Further work is summarised in Table 1 below for case studies involving water quality. Results based on these findings are inadequate and biased since dissolved chemical pollutants, like organic compounds, also contribute to water contamination, an observation highly common in areas with intensive agricultural activities [58,59]. This underscores the inclusion of pesticide data during water quality assessments. Studies have, however, highlighted lab requirements and sample contamination as major challenges in detecting organic pollutants, hence making pesticide monitoring unpopular [60,61]. Despite these hurdles, pesticide-contaminated water presents considerable health hazards making it essential to monitor its occurrence in water sources. Furthermore, valuable insights into the actual network performance can be inferred by modelling the signal propagation and enabling optimisation strategies that can enhance efficiency and reduce deployment costs. Therefore, this study aims to investigate pesticide contamination in water bodies while simultaneously assessing the LoRa signal propagation to improve the network design and implementation.

## 3. Field Study Site

**Figure 1:**
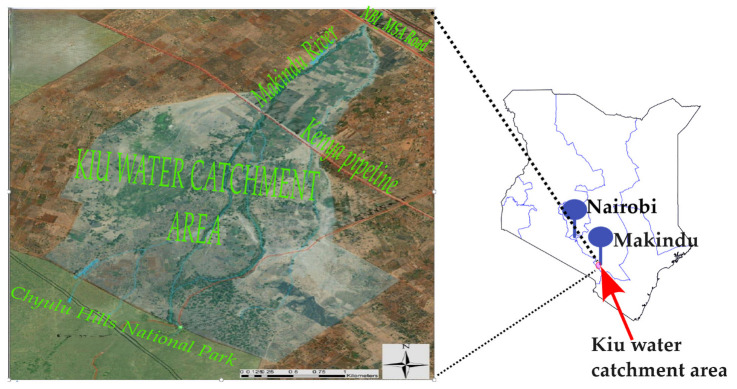
A map of the Kiu wetland.

The Kiu wetland lies in Makueni County, an arid and semi-arid zone experiencing a tropical climate with two rainy seasons and well-defined dry conditions from June to September. It is located on the western side of the Makindu market, approximately 3 km from the shopping centre and about 170 km southeast of Nairobi, the capital city of Kenya. The catchment area occupies an expansive landmass stretching from the Nairobi–Mombasa highway up to the Chyulu Hills National Park. The area is relatively flat on the northern and southern sides, but the terrain greatly changes on the west side bordering the national park and a deep basin across the middle. A seasonal river, the Makindu River, shown in blue in Figure 1, cuts across the watershed, providing water for agriculture and domestic use.

The Kiu wetland doubles as a water catchment area and arable land planted with different horticultural crops. The water tower benefits from its nearness to Chyulu Hills, which receive a slightly higher than average rainfall. Most parts are covered by the volcanic black soil, which turns sticky and waterlogged during rainy seasons. A high water table and fertile soil make it attractive for small-scale farmers to practice agriculture, whose farms are interspersed with thick tree canopies.

**Figure 2:**
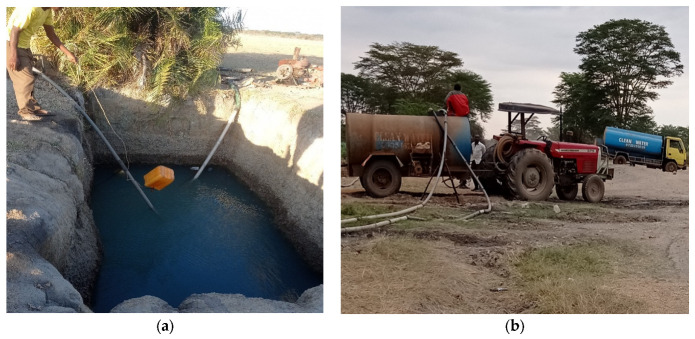
(**a**) Shallow well. (**b**) Refilling water bowsers from shallow wells.

The continuous growth of crops with minimal crop rotations makes pests and diseases difficult to control naturally; hence, pesticides are uses as a control mechanism. When the river flow reduces, farmers are compelled to sink shallow wells for irrigation during dry seasons. Vumilia village lies on the extreme end near Makindu market. Its strategic location near to the urban centre means that it enjoys an improved infrastructure and hence is an attraction to many farmers who find it easy to access the market for their produce. This significant population leads to the sharing of shallow wells, shown in Figure 2a, by a number of homesteads, making it possible to apply the monitoring system developed. Additionally, water bowsers, as seen in Figure 2b, can easily access the wells for refilling to distribute water to the neighbouring regions. The clearly labelled containers indicating clean water are sufficient to assure potential consumers of its quality, minimizing concerns about its source.

## 4. Materials and Methods

### 4.1. Radio Planning

LoRaWAN operates in different frequencies worldwide, with additional regional regulations as provided by The Things Network. However, some countries like Kenya neither have the frequency assignment nor the regulatory framework for LoRaWAN networks [70]. Various models were evaluated to establish how accurate they predicted the data observed in the area under investigation. Emphasis was put on models utilised in LoRa propagation in rural areas like our study area. As the region lacks an existing IoT ecosystem, a custom gateway was designed for the coverage of sensor nodes.

#### 4.1.1. Propagation Models

According to the Friis equation, decay in energy is a function of distance between the transmitter and receiver. However, this theory holds true in line-of-sight idealised situations. In real-life scenarios, the expanding signal undergoes attenuation due to clutter on its path and environmental factors. The propagation channel of a signal is often assumed to be in elliptical shapes commonly referred to as Fresnel zones. For optimal performance of LoRa, the first Fresnel zone, where signal energy is most concentrated, must be kept approximately 60% clear [71]. Deterministic models, built on strong analytical foundations, using laws of physics and specific environmental factors and obstacles [72], are particularly suited for scenarios where detail and accuracy are required. The FSPL model is given in Equation (1) below, and ray tracing belongs to this group, where the former is used as a foundational reference point for path loss considering LoS conditions [73,74].

(1) $$PL=20\log_{10}d+20\log_{10}f+32.4$$

where f is in MHz and d in kilometres.

Signal attenuation is influenced not only by large, rigid obstacles but also by smaller particles and the terrain, both of which contribute significantly to signal degradation. Small particles, which are in transient motion, cause diffraction or slow fading, while attenuation caused by larger obstacles is typically referred to as fast fading or shadowing [75]. The other approach, known as empirical modelling, is derived from observed data and statistical properties, often incorporating adjustments based on measurements specific to the environment. A common correction for FSPL to cater for NLOS is to use a large exponent [76].

(2) $$PL=10\gamma \log_{10}d+20\log_{10}f+32.4$$

where f is the frequency in MHz and d in kilometres.

The path loss exponent γ is 2 for FSPL but can vary significantly ranging from 2 up to 8 depending on environmental setups [77].

- Log-distance path loss model

The model is based on assumption that path loss increases with distance in a predictable logarithmic fashion according to the following equation [78].

(3) $$PL=PL\left(d_{0}\right)+10n{log}_{10}\left(\frac{d}{d_{0}}\right)$$

n—path loss exponent (can vary between 2 and 6 depending on the environment [44]), d—separation distance between receiver and transmitter, and d_0_—reference distance can be between 1 and 10 m for microcell and 1 km for large cell. It is obtained by regression fitting of data from measurements.

- Okumura–Hata

Hata model established mathematical equations for a set of curves from Okumura’s extensive measurements results of base station-mobile unit attenuation in Tokyo, Japan [79]. This formulation made it easy to analyse propagation losses as calculation was not based on graphical plots. The model is valid between frequency ranges of 150–1500 MHz and a link distance of 1–20 km. Since the LoRa radio used in this research operates at 915 MHz it falls well within the applicable frequency range of the model. The expressions are appropriate for nearly flat terrain with an applicable user antenna height of 1–10 m and base station antenna height of 30–200 m. The original design was for urban areas but has corrections for suburban and rural areas as well [80].

(4) $$P_{L_{Urban}}=69.55+26.16\log_{10}f-13.82\log_{10}h_{B}-A\left(h_{m}\right)+\left(44.9-6.55\log_{10}h_{B}\right)\log_{10}d$$

where $h_{B}$—height of base station antennae (m), $h_{m}$—height of mobile equipment antennae (m), f—frequency (MHz), $A\left(h_{m}\right)$—correction factor for mobile antennae height, and d—distance between base station and mobile equipment (km). Three adjustment factors are applicable; however, given that this research is conducted in a rural setting, the most relevant environmental adjustment is presented below.

(5) $$A\left(h_{m}\right)=(1.1\log_{10}f-0.7)h_{m}-\left(1.56\log_{10}f-0.8\right)$$

Path loss in open/rural areas is given by

(6) $$P_{L_{Rural}}=P_{L_{Urban}}-4.78{(\log_{10}f)}^{2}+18.33\log_{10}f-40.94$$

- COST 231-Hata

The model is an extension of the Hata model to extend the frequency range up to 2000 MHz [81]. Path loss predictions are based on the following parameters: a distance of separation of 1–20 km and an antennae height of 1–10 m for end devices, whereas for base stations it is 30–200 m. Its mathematical formula is as follows, with corrections for urban, suburban, and rural areas [82]. The rural correction supports the validity of the model in our study.

(7) $$P_{L}=46.3+33.9\log_{10}f-13.82\log_{10}h_{b}-a\left(h_{m}\right)+\left(44.9-6.55\log_{10}h_{b}\right)\log_{10}d+C_{m}$$

where f—frequency MHz; d—link distance in km; h_m_—mobile station antennae height in m; C_m_—constant, 0 dB for open environments/suburban and 3 dB for urban environments; and h_b_—base station antennae height in m. The parameter a (hm) for environment is defined as [83]

Urban correction

(8) $$a\;(h_{m})={3.2(\log_{10}\left(11.75h_{m})\right)}^{2}-4.97,\;f\;>\;400\;\mathrm{MHz}$$

Suburban/rural

(9) $$a\;(h_{m})=(1.1\log_{10}f-0.7)h_{m}-\left(1.56\log_{10}f-0.8\right)$$

#### 4.1.2. Forest Terrain

Signal propagation within a foliage medium is complex due to the interaction between electromagnetic waves and the discrete scatterers present in the vegetation [84]. These interactions give rise to various phenomena, including scattering, diffraction, and absorption, all of which contribute to substantial signal attenuation and fading [85]. Other dynamics of path loss are introduced by the structure of vegetation and the effect of environmental factors like wind, rain, humidity, and temperature [86,87]. The area under investigation is forested which implies greater signal degradation compared to environments assumed in models based on rural open-area conditions. The presence of tall trees, extensive branching, and dense ground vegetation suggests that signal propagation is primarily through the canopy layer. In previous studies this has been reported to result in higher attenuation levels than propagation through the trunk region [88,89].

#### 4.1.3. RSSI and SNR Measurements Across Deployment Area

**Figure 3:**
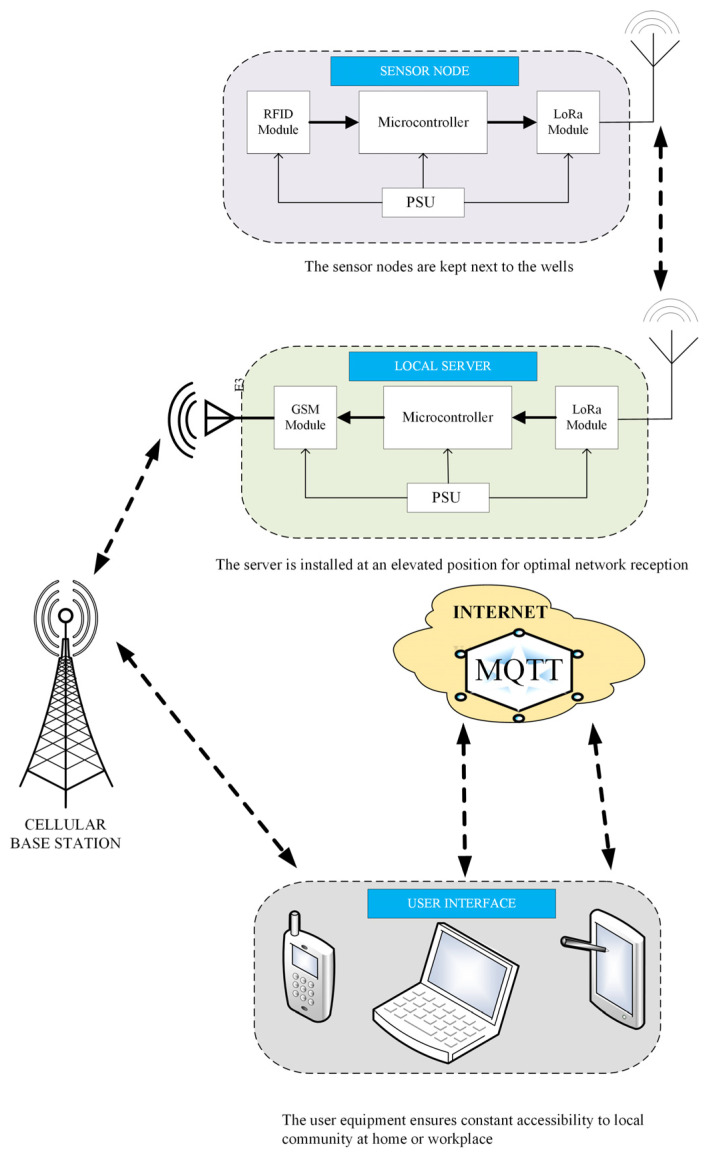
IoT-based WSN system for pesticide monitoring.

A custom-made server featuring a gateway was designed to allow sensor nodes to forward packets from the field for processing. An Arduino Uno R4 (Arduino S.r.l societa unipesonale, Piedmont, Ivrea, Italy) is interfaced with a Semtech 1276-based HopeRF LoRa chip (Shenzhen Hope Microelectronics Co., Ltd., Shenzhen, Guangdong, China), with sensitivity down to −136 dBm [90] and an SD card for logging the packets. Internet connectivity was enabled via GPRS for seamless visualisations of environmental data by users through their mobile phones and computers. The cellular module also sends SMS to users when pesticide determination of various wells is complete. Figure 3 below gives a layout of the communication between various modules. The RFID module in the sensor node feeds pesticide names into the microcontroller after tests are performed utilising paper-based sensors. These packets are then forwarded to the server via LoRa protocol by pressing push buttons on the LCD interface.

The first phase of the experiment aimed to determine how LoRa signals varied spatially across all wells in the wetland. To achieve this, LoRa metrics were collected from every identifiable well within the area. To evaluate the channel quality, the receiver was propped on a pole in an upright position then secured to a tree fastened with ropes for good anchorage. Link characteristics were tested with 2 LoRa chips with identical spiral antennae of 2.15 dBi gain. The receiver was fixed 5.1 m above the ground to improve reach, while the mobile unit was attached to the fuel tank of a motorbike. This unit was configured to send an 11-byte payload and then listen for an ACK from the receiver with 4 millisecond timeouts. The process was set to repeat until an ACK was received, which would be followed by sending an additional 49 packets of the same payload size consisting of RSSI and SNR values. The transmitter was allowed to send packets after reaching a shallow well with other details, including coordinates recorded using Google Maps. The design for the transmitter and receiver is according to the description explained in [91].

### 4.2. Water Contamination Measurement

#### 4.2.1. Measurement Campaigns and the Feeding of Data into the Sensors

The second phase of the experiment focused on monitoring pesticide prevalence in three selected wells within the wetland over an extended period. The objective was to assess the contamination status of the water at the time it was drawn for domestic use. Out of the 21 wells investigated for radio propagation, only 3 were required for the pesticide measurements, a limitation imposed by the number of developed sensor nodes. The selection was not limited to those identified for radio planning as long as the well was within the mapped region and had the desirable characteristics. The qualifying criterion for inclusion was that a well was required to have multiple users to enable the system to send bulk messages on events of pesticide monitoring. Preference was given to wells with the highest number of users who possessed a mobile phone and were willing to participate in the study. Wells owned by single users were therefore excluded from study as the system was not applicable. To ensure anonymity of data, unique participant codes were adopted as follows: PxxxY for those who took part in the exercise.

Where

P—participant;

xxx—serial of participant;

Y—well identity.

Using this naming taxonomy, the following patterns provided in Table 2 were generated.

- Human–system interaction

In the final phase of the experiment, citizens were expected to eventually input data into the sensor nodes. To evaluate the usability of this process, a group of individuals was selected to test how easily they could enter data into the developed sensor nodes. Their input times were also recorded as part of the assessment. A random selection of 9 participants from the local community was carried out, labelled from V001 to V009. The participants were drawn from various age groups and educational backgrounds. Each was taken through the process of data entry independently in a controlled manner by using a single NFC tag. The aim was to analyse the time each participant took to interact with the developed user interface in sending a packet across six non-consecutive trials.

#### 4.2.2. Sample Collection and Pesticide Determination

Samples for testing and screening of pesticides were collected 0.5 m below the water surface. Sampling was performed once every day with timings guided by the well users’ domestic water demands.

To conserve the time for loading data to the system, the sensors were placed next to the wells selected for monitoring. After completing the test for a certain sample, the results were uploaded without delays. The sensors are paper-based, a choice made on account of economic factor, ease of use, and specificity. No sample pre-treatment procedures were carried out for the field tests.

- Pesticide residue tests

**Figure 4:**
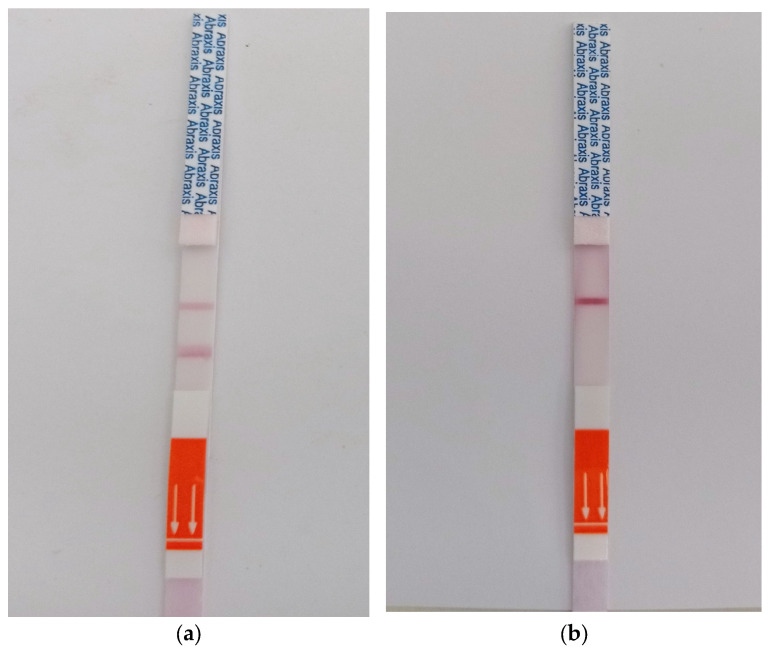
(**a**) Test result of shallow well water showing test and control lines. (**b**) Test result for shallow well water spiked with glyphosate. Arrows indicate direction of dipping in a sample.

Gold Standards Diagnostics (Warminster, PA, USA) sensors were used to test for the presence of pesticides in the selected wells after sample collection. These sensors consist of a window for observing the colour change upon addition of a pesticide on the reaction pad. The antibodies in the sensor bind with the pesticide, which can easily be noted by appearance of coloured line on the window shown in Figure 4a. The concentration of the pesticide is then inferred by comparing the control line to the test line, as shown in Figure 4b below.

**Figure 5:**
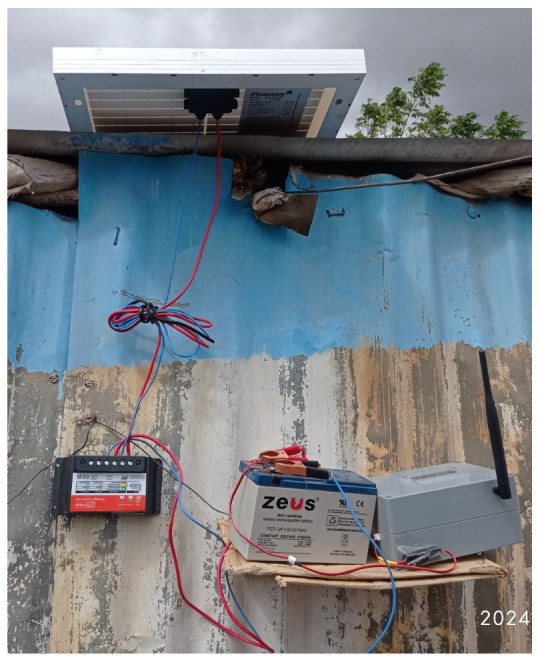
The server.

The gateway was suspended, as shown in Figure 5, in elevated position 2.3 m above the ground in a compound with iron sheets perimeter wall. This enabled effective reception for data packets from the field as performance reliability in LoRa increases with antenna height [92]. In addition, perimeter walls provided protection against humans and animals. It was positioned beneath the solar panel to protect it from adverse weather effects, particularly to limit the rise in battery temperature and prevent thermal noise from radiant heat.

**Figure 6:**
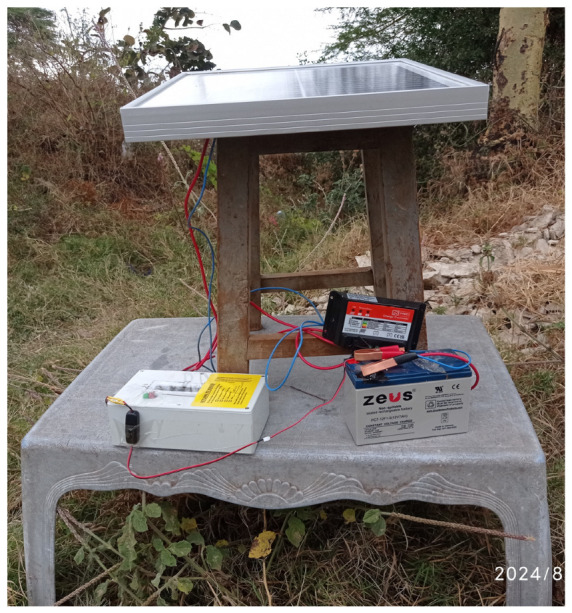
A sensor node setup for capturing data.

A solar charging system was designed, consisting of a solar panel, a charge controller, and a battery, as indicated in Figure 6 below. The system ensured sustainable measurements and uninterrupted data uploads as typically observed during power outages.

**Figure 7:**
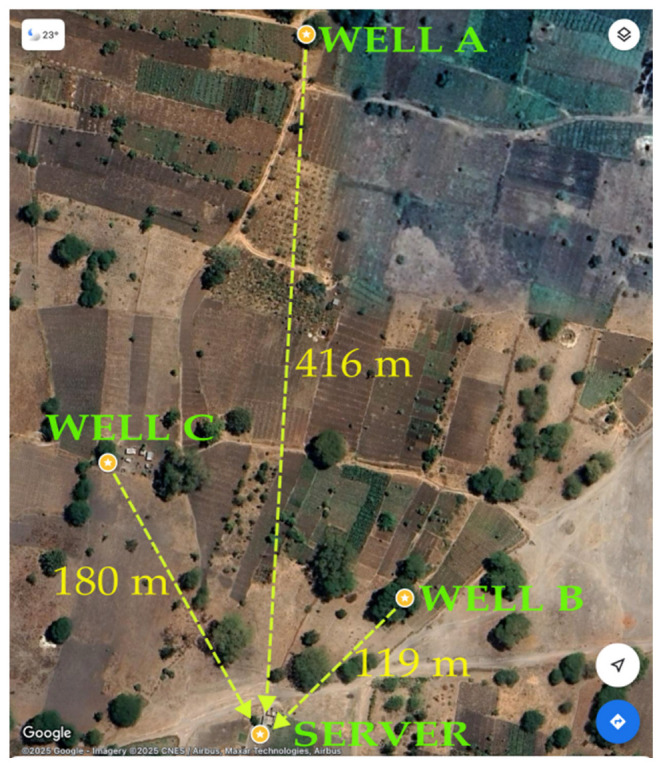
The measuring distance from the wells to the server. Well A = 416 m, well B = 119 m, and well C = 180 m from the server.

Identification of the device location relative to the ground was performed using Google Maps, and the following layout in Figure 7 was observed. Distance from the server was determined using the distance measurement feature offered by Google Maps.

## 5. Results

### 5.1. Simulation Analysis

The analysis of the simulation was performed using MATLAB Version: 24.1.0.2689473 (R2024a) Update 6 software. The evaluation of the model performance was achieved by considering the mean absolute error (MAE), the root mean square error (RMSE), and the standard deviation (SD), whose details are provided in Table 3.

#### 5.1.1. Mean Absolute Error (MAE)

The MAE is a quantity that gives the average of errors irrespective of the sign. It indicates how close the predicted RSSI values are to the actual field measurement and hence provides a direct measure of the model’s performance. The lower the MAE value the better the agreement. From the results, log-distance was the most accurate model with the lowest MAE of 5.1611. Both the Okumura–Hata and FSPL values show a significantly large deviation from the field data.

#### 5.1.2. Root Mean Square Error (RMSE)

The RMSE is a metric which quantifies the average deviation of a model’s prediction from the actual values with a higher sensitivity for large errors. It is calculated as the square root of the average of the squared differences between the predicted values and the field data. Among the propagation models tested, the log-distance model demonstrated the least deviation from observed values, with R = 6.2493. A lower RMSE value indicates better model accuracy, with acceptable values in the range of 6–7 dB [93]. COST 231-Hata shows a relatively good performance compared to the rest but with slightly more errors than log-distance.

#### 5.1.3. Standard Deviation (SD)

The SD is the square root of the variance, which shows the spread or variability of the data across the observations. Apart from FSPL all the other models posted nearly equal values of the standard deviation, an indication of the similar variability of prediction errors. RSSI values predicted by FSPL show the lowest SD, indicating consistent errors across the measured distances. However, its predictions are systematically optimistic compared to field measurements, with an average bias of +42 dB. To confirm its claim, its position should be farthest from the curve of best fit. From the analysed metrics, the log-distance model best predicted the field RSSI values.

### 5.2. Experimental Results Analysis for Radio Planning

#### 5.2.1. Layout of Wells

**Figure 8:**
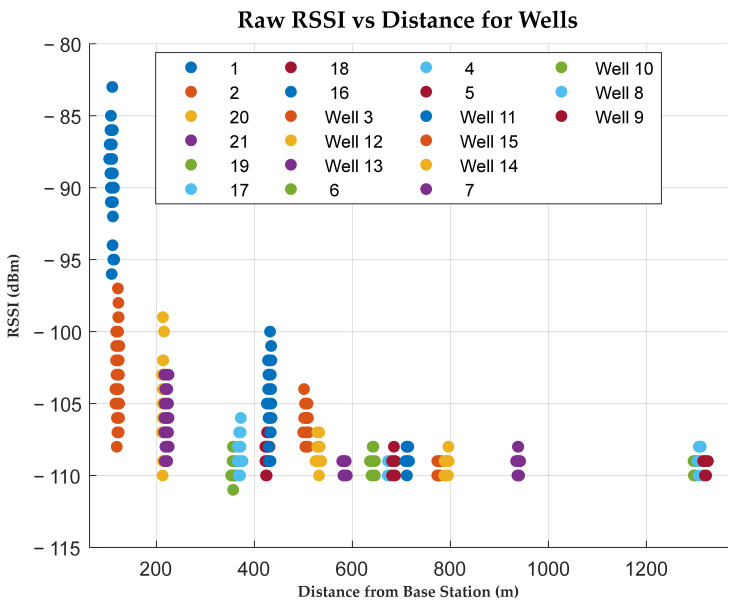
A plot of RSSIs across wells ordered by the distance from the server. Coloured blobs indicate raw RSSI values. Sites near the server have a greater variation in RSSI values, but their convergence occurs as the distance from the server increases.

**Figure 9:**
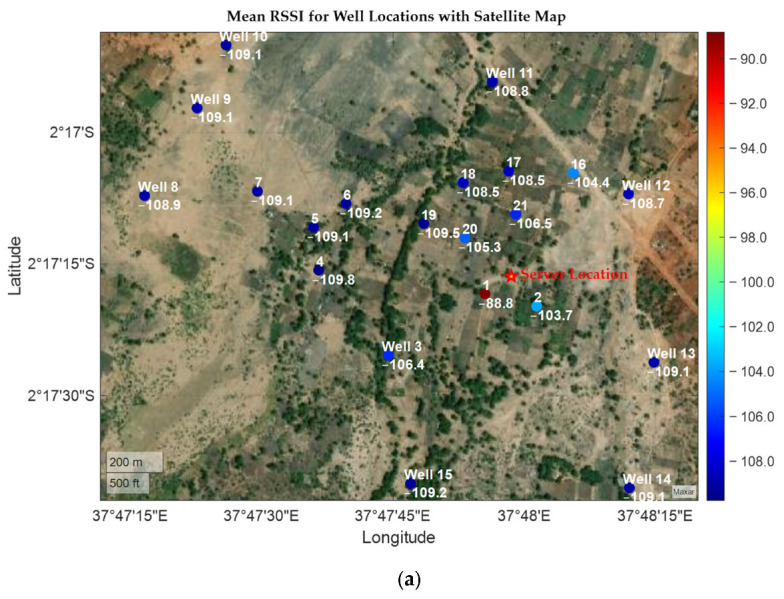

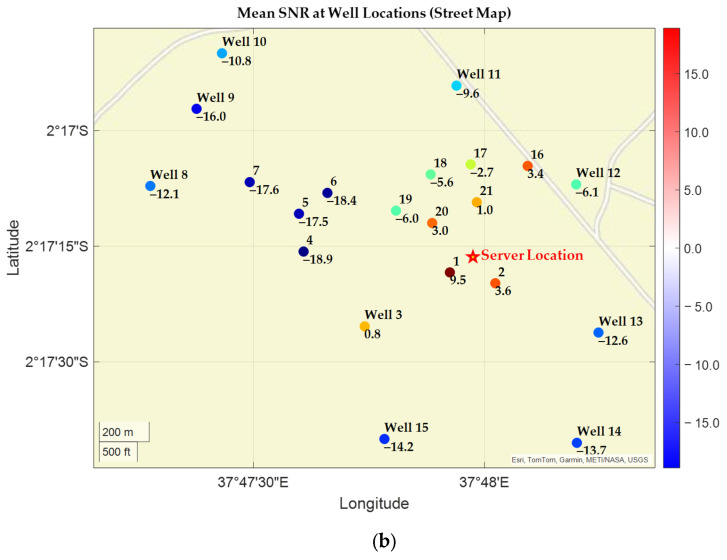
(**a**) A heatmap of the well layout with RSSI values. (**b**) A heatmap street-view of the well layout with SNR values.

From Figure 8, the wells located near the server had higher RSSI values compared to those far away, this was expected as the RF energy decreases as one moves away from the source. Their average RSSI values were in the range of −88.83 dB to −108.50 dB; however, discrepancies were noted in well 19, which despite its nearby location had a relatively lower RSSI. This was attributed to the unique terrain surrounding it. Being in a river basin, the ravines limited the smooth RF propagation to reach the server, hence affecting the signal strength. Similarly, wells 4, 5, and 6 recorded lower RSSI values due to the surrounding clutter, as seen from Figure 9a. The wells are located right inside a thick canopy of tall trees with numerous branches, which affect the signal quality. A low packet success rate for the three wells further explains the effect of obstructions on the communication link. An improved strength is noted in well 7, as it is located in an open ground after the forested area. Out of the 21 wells investigated, 17 posted a packet success rate of more than 80%; this represents 81% of the total wells, demonstrating the system’s reliability in the packet delivery and gateway coverage [94]. Well 20 recorded the highest packet delivery success rate of 98%. Its nearness to the server placed it at an obvious strategic point for a better performance than others. However, this points to the need for contingency plans during sensor deployment to establish a loopback or acknowledgment mechanism to minimize chances of notification failures. As observed in Figure 9b, wells farther from the server recorded lower SNR values, often dropping below −10 dB, indicating the increased environmental noise and signal degradation. This compromised the transmission integrity, resulting in mutated or incomplete packets that hinder the server-side interpretation of the received packet and SMS notification. However, attaching a high gain antenna with a 3–6 dBi gain can produce an enhanced signal strength over greater distances, minimizing payload errors.

#### 5.2.2. Monitoring of Pesticide Residues

In all the measurements carried out during the monitoring period, no detection of pesticide residues was noted. While the data collected during the coverage analysis had comparatively higher values of the RSSI, the daily packets registered significantly low values as a result of the receiver height reduction from 5.1 m to 2.3 m. Well B, which was 119 m away from the new location of the server, registered the highest RSSI mean of −110.63 dB, with A and C postings of −113.91 dB and −113.24 dB, respectively. A further investigation shows the plateau behaviour of the LoRa signal after reaching a certain minimum, with the RSSI means of A and C being very close despite the two having a separation distance of 236 m. The reduction in the height of the server caused the possible obstruction of the first Fresnel zone, impacting the transmission by weakening the signal strength. Additionally, suspending the server within sheet metal walls further scattered the radio waves, deteriorating the quality of the reception even though no formal experiments were performed to investigate the overall effects.

**Figure 10:**
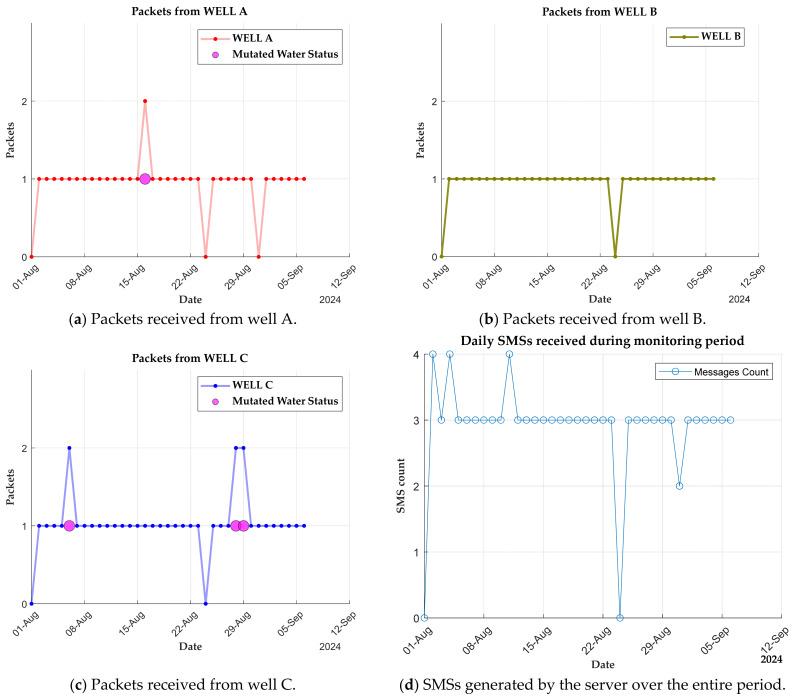
Packets received by server from sensor nodes and messages sent to users: (**a**) well A, (**b**) well B, (**c**) well C, and (**d**) SMSs generated by server.

In total there were 15 instances of mutated payloads over the monitoring period, and 11 of them were related to well names and the rest water status. These packets contained unknown characters, missing letters, empty strings, or altered patterns. Out of the four cases of a wrongly captured water status, three of them originated from the sensor node placed in well C, and one was from well A, as observed in Figure 10. Such payloads could not be recognised as valid packets by the server, and as a result a corresponding SMS could not be generated. Details from Figure 10b indicate that the sensor node in well B did not transmit any packet in a wrongly written format. Packets from well A and well C, being farther away from the gateway, may have experienced signal degradation, leading to their deformation. A higher signal attenuation is observed in packets sent form well C, as evidenced by a higher percentage of mutated cases compared to well A, despite being at a nearby position. By including a feedback mechanism, the system can detect when messages are not delivered, which leads to resending the packet. This improves the reliability and reduces the risk of missing pollution events.

Every day, the system generates one message after receiving a LoRa packet from a particular sensor node. Figure 10d below is an analysis of a SMS received by a user registered in the three wells. It provides the total messages that the system sends to a user in a day. These messages are composed in the local dialect to enhance information accessibility and comprehension—a deliberate mechanism aimed at reaching those intended in the community. In addition, the graph indicates the presence of other SMSs in various days. These were meant to report the health status of the server after restarting; however, the general public are forbidden from receiving them.

The community user input timing study showed that the average time for all participants in feeding the data dropped significantly from the first trial to the last trial. Generally, the average time for all participants in trial 1 was around 30 s, while by trial 6, it had dropped to about 18 s. Further insights from Table 4 show that education and age did not seem to influence the time taken by the participants in feeding data to the sensor, an indication that once an individual is taken through the procedure, they can equally participate in inputting the data regardless of their age or educational background, and only the individual variability was a significant factor.

#### 5.2.3. Validation of Pesticide Residue Results Using Laboratory Procedure

Three samples labelled Well A (ID AE 240005), Well B (AE 240006), and Well C (AE 240007), collected from the respective wells, were screened for pesticide residues and polychlorinated biphenyls using LC-MS/MS and GC-MS/MS procedures. The lab results indicated that no analytes were detected, with a Limit of Quantification (LOQ) of 0.01 mg/kg. A list of the analytes considered for the screening process is provided in Appendix A. From the calibration curve, a linear regression model was applied for glyphosate with the equation

(10) y=1.302934x−0.013654

**Figure 11:**
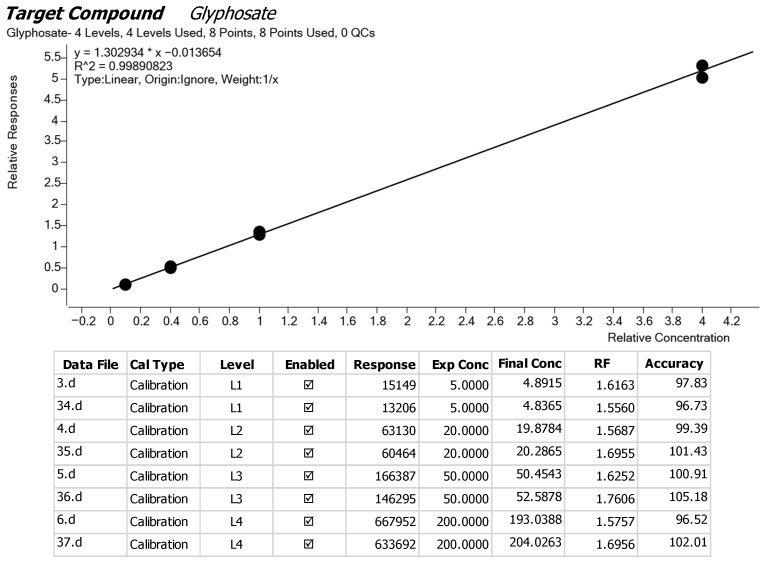
Glyphosate calibration curve.

The value of R^2^ = 0.99890823, suggesting that the data fits well in the linear model, as seen from Figure 11. Different standard concentrations of glyphosate were used in the calibration, as evidenced in levels L1–L4. For the low-level calibration L1(3.d), the final concentration is 4.8915 ppb for a 5 ppb standard with a 97.83% accuracy, indicating a slight underestimation. Similar results are observed in file 34.d, whose accuracy is 96.73%. L3(5.d) demonstrates an accuracy of 100.91%, which is quite close to the expected value. However, there is a significant fluctuation at the higher levels L4(6.d) and L3(36.d). In general, glyphosate demonstrates a relatively consistent accuracy across all levels (around 96.52–105.18%), suggesting reliable calibration.

**Figure 12:**
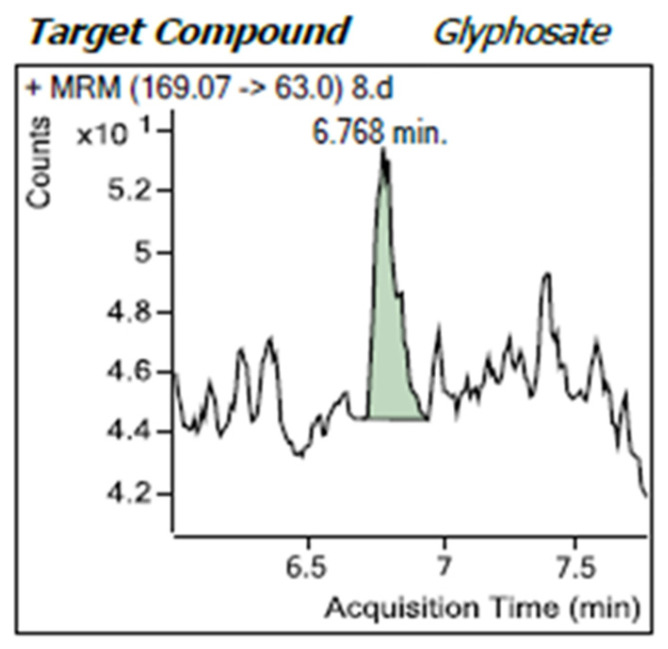
A graph of the intensity of the detected signal versus the retention time. Small fluctuations indicate baseline noise ranging from 43 to 48 counts with a peak intensity of 54 counts at elution.

**Figure 13:**
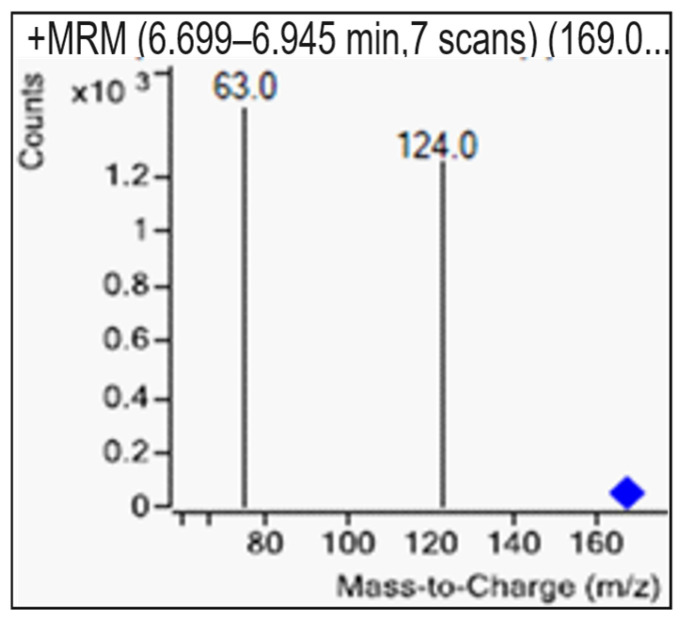
A graph of the fragmentation pattern of glyphosate, showing a precursor ion (blue diamond) and 2 daughter ions.

**Figure 14:**
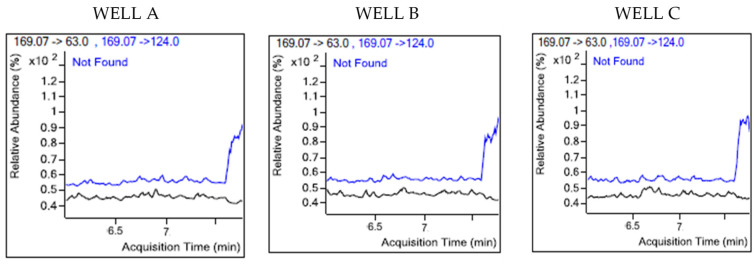
Graphs of monitored transitions for wells A, B, and C over 2 min acquisition time.

The mass spectrometry analysis using multiple reaction monitoring was performed on samples, as illustrated in Figure 12, with glyphosate eluting at 6.786 min. The chromatograms displayed sharp peaks corresponding to glyphosate and its metabolites. Figure 13 shows that the precursor ion and daughter fragments were targeted at mass transitions, mz169→124 and mz169→63. The presence of glyphosate was not detected in the three samples presented for screening, as indicated in Figure 14. Both blue and black traces have been marked by the system as “*not found*”, suggesting no measurable compound at these transitions. The results were in total agreement with the field tests performed on 28 August 2024 when the samples were collected.

## 6. Discussion

### 6.1. LoRa Signal Propagation in the Wetland Terrain

**Figure 15:**
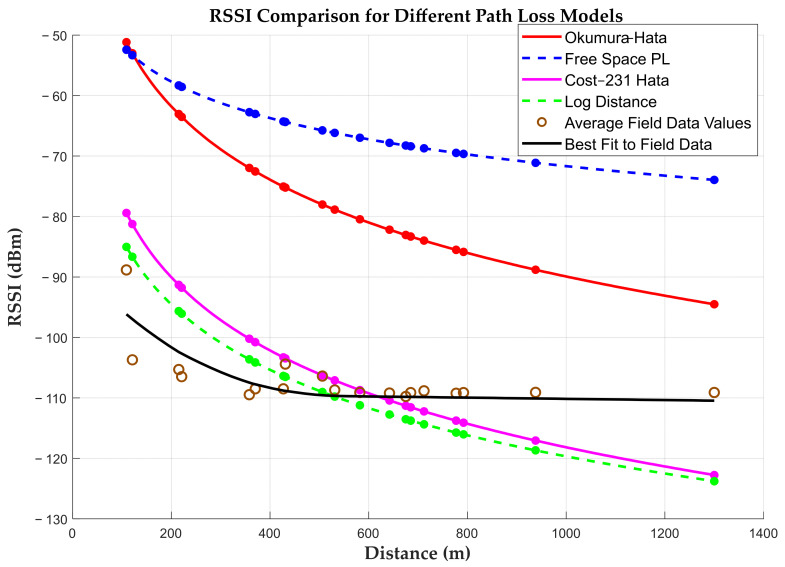
A graph of path loss models.

Our results from the field, shown in black in Figure 15, were compared against other path loss models using LoRa radio settings applied in the data collection and respective antennae heights. The frequency = 915 MHz, the height of the base station = 5.1 m, the height of the mobile unit = 1.0 m, and the transmit power = 20 dB. For the COST 231-Hata model, the C_factor was taken to be zero for the rural environment, while in the log-distance model the reference distance was d_0_ = 1 and n = 3.6 [95]. The visual observation of the curves indicate that all the model curves dipped lower with the increasing distance, while the field data maintained a plateau with a small negative gradient after reaching a minimum point at 568.16 m.

The accuracy of the models was strongly influenced by the distance from the gateway. Insights into the signal loss behaviour before the plateau and after the plateau indicate a non-linear and more chaotic degradation near the gateway. This abrupt drop in metrics near the gateway is common in other studies [96]. A comparison of MAE/RMSE values for the models, before and after the plateau, is as follows: FSPL (44.14/40.58, 44.14/40.62), Okumura–Hata (36.8/24.65, 37.1/29.94), COST 231-Hata (8.55/3.83, 9.74/5.21), and log-distance (4.77/5.59, 6.00/6.51). With the exception of the log-distance model, the trend in all the other models demonstrates a decay in the near-field region. LoRa uses chirp spread spectrum modulation and performs well in long-range, low-SNR environments, which is typical of what the region experienced after the plateau, where the signal is weak but stable [97]. The steep decay in RSSI values is attributable to the fast loss in near-field antennae radiation patterns and environmental obstruction, which make it difficult to model accurately, hence experiencing pronounced errors [40]. The log-distance model shows a slightly higher error after the plateau, which makes sense since it models the path loss logarithmically, assuming a relatively consistent behaviour which gets disrupted when the RSSI stops decaying as much in the plateau region. The stability of RSSI values after the plateau is likely due to the limited multipath and stable air path with low obstacles. The Okumura–Hata model’s assumptions of higher base stations and user heights were not achieved in this study since the transmitter was placed 5.1 m above the ground, violating antenna requirements. The choice of this height was guided by allowable safe heights in the area under study. COST 231-Hata, designed for rural environments, performs better after the plateau but could not accurately model near the gateway, as the interference is more variable. Log-distance seems to adapt better to the RSSI sharp drops and gradual decay, suggesting its suitability in a mixed terrain, like the one encountered. These dramatic differences demonstrate that traditional propagation models which assume a continuous signal decay with distance fundamentally cannot capture the plateau phenomenon observed in our measurements. This analysis suggests that when deploying LoRa radios in similar environments, traditional propagation models should be modified to account for this signal stabilization at longer distances.

### 6.2. Influence of Weather Conditions on Pesticide Residues

#### 6.2.1. Pre-Monitoring Period

With an average temperature of 28.1 °C, July’s temperatures were warm enough to favour the pesticide degradation through thermal processes. There was no rainfall throughout July. Without rainfall there was no leaching or runoff of pesticides into the soil or water bodies. The lack of rainfall means no transportation of pesticides to other areas. Wind speeds in July were moderate, ranging from 2.5 km/h to 5.8 km/h. This could have contributed to the pesticide drift or the dispersion of any applied pesticide into non-target areas away from monitoring sites. Historical weather data helps explain the occurrence of slow processes like leaching which affect the migration of pesticides in the environment.

#### 6.2.2. Monitoring Phase

**Figure 16:**
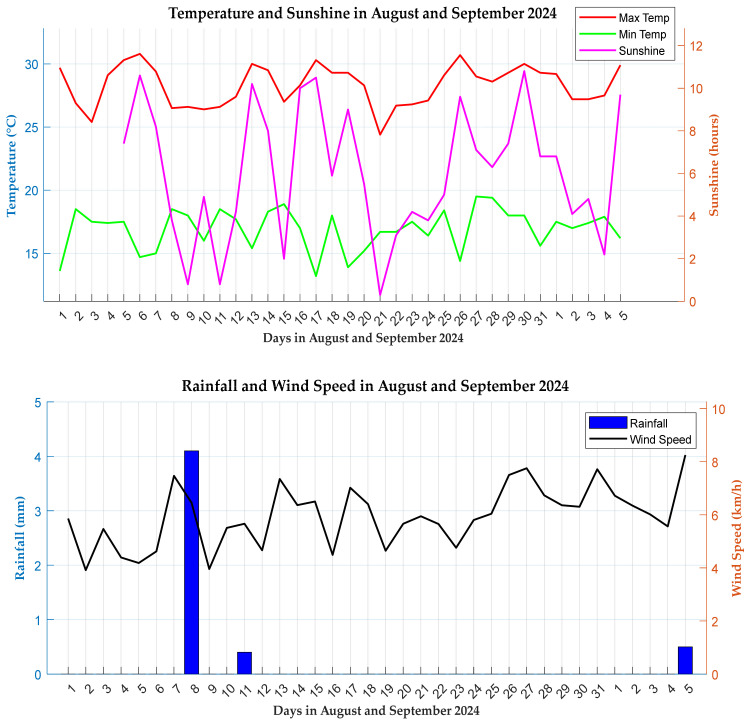
Weather graphs over monitoring period.

The weather conditions in August and September show relatively high temperatures averaging 28.3 °C and a sunshine duration of 6.06 h, as provided in Figure 16. Both factors promote the degradation of pesticides. High temperatures can increase the rate of reaction, leading to the faster breakdown of pesticides through volatilization. Sunshine, specifically UV light, accelerates degradation through sunlight-induced photolysis. This combination may have effectively degraded pesticides before they could be detected.

The rainfall data shows a minimal precipitation average of 0.14 mm across August and September. A low rainfall reduces the likelihood of pesticides leaching into deeper soil layers or the runoff to nearby water bodies. Rainfall is a key factor in pesticide transport, as its low levels indicate limited movement within the environment, reducing their presence in sites where they would otherwise accumulate. Similarly to July, August and September experienced relatively moderate wind speeds, averaging 12 km/hr, blowing away any applied pesticides to other regions.

### 6.3. System Usability Analysis

The use of SIM 800 in the project demonstrated its ability for reliable communication in rural areas for SMS alerts and MQTT data publishing via HiveMQ. The GPRS connectivity was achieved within 10–13 s from a cold start with the APN set to the local mobile network even in areas with a GSM signal strength of −107 dB. Once a LoRa packet was received from a field device, data was published in the MQTT near real-time to topic structures formatted as follows.

“Date”: 6 December 2024, “time” 10:56:18, “topic”: mutunga/chemicals, “well ID”: WELL C, “pesticide”: carbaryl, “RSSI”: −42. No publish failures were noted once the MQTT broker was connected; however, the module required reconnection attempts during the GPRS outage, with the events being self-recovering and infrequent.

For SMS functionality, messages were issued to users after the server received a valid packet. A delay of 5 s was introduced to allow the module to process the SMS to be sent as the module was observed to be slow. This delay was increased to 7 s for a list of phone users per well in the range of 5–12. The SMSs were formatted as follows “well ID” WELL A “sms”: *maji ya kisima chako ni safi* (the water in your well is clean) or *maji ya kisima chako ni sumu hatari* (the water in your well has a dangerous poison). User’s feedback informed the change in format from an earlier SMS version, *maji safi* or *sumu hatari*. Although the messages meant clean water and a dangerous poison, respectively, the users suggested that the messages could not be well understood as they were too brief, prompting the change to the new format. Successful transmission reports were received on all target devices without duplication. Using a dual communication strategy featuring MQTT streaming and SMS alerts was an effective redundancy measure in the rural deployment where the infrastructure is limited.

To facilitate easy and non-expert methods of data entry, near-field communication (NFC) was implemented for loading the pesticide data directly into the memory of each sensor node. An NFC module bearing the PN532 chipset was integrated into sensor nodes and interfaced with the microcontroller via I2C. This feature allows field personnel to capture correct names of pesticides without requiring a keyboard, which might introduce typo errors. In addition, an LCD shield bearing push buttons was also included in the design to allow users to interact with sensor nodes, utilising prompts of visual cues on the display to input the field data. Once a pesticide is detected, an NFC tag is swiped across to transfer the name of the pesticide into the sensor node, generating the LoRa packet for transmission. This interface also allowed users to conveniently cancel any operation they suspected may be incorrect, minimizing any errors in the data collection exercise. During the data transmission an LoRa packet is sent to a gateway, which also functions as a local server.

**Figure 17:**
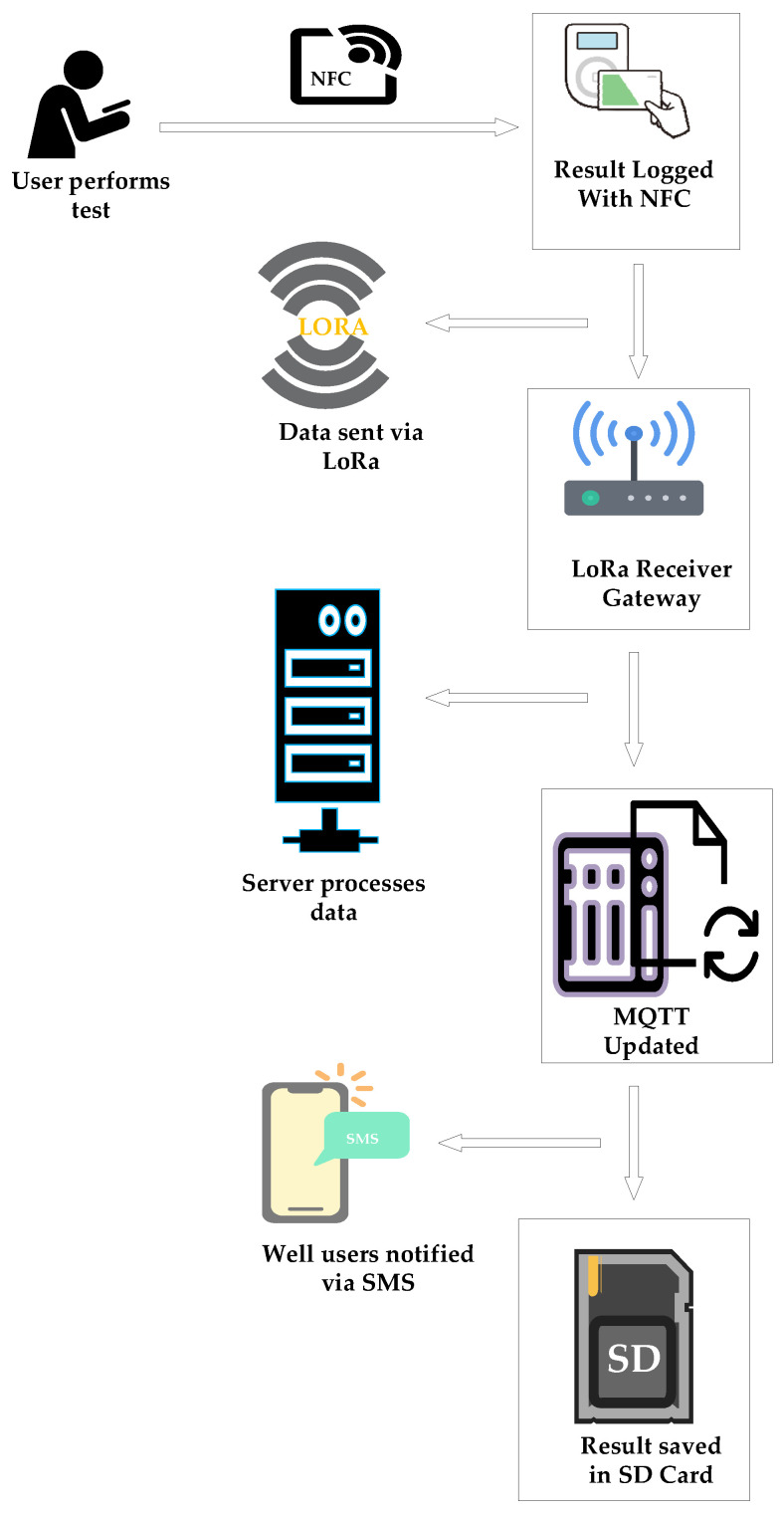
Process flow chart for pesticide sensing.

The packet processing of the field data begins by checking whether it contains all essential components, including its origin, contamination status, and RSSI value. The identification of the well and its corresponding condition informs the selection of the registered users and the determination of the SMS to be multicast. These portions are passed to a subroutine responsible for polling all the members associated with the identified well for an SMS. In parallel, an additional notification is disseminated via the MQTT portal to cater to users with internet-enabled devices. To ensure the completeness of the visualisation, all the three components of the field packet are utilised by the function handling the MQTT publishing. As a precautionary measure, this function includes a verification process for the status of the GPRS connection, with automatic reconnect attempts in case of outages. Upon a successful connection, the message is forwarded to the Hive broker for publishing. To maintain data availability and facilitate an offline analysis, the LoRa packet is logged onto an SD card. Prior to storage, a timestamp is fetched from the NTP servers and then prepended on the packet before it is written on the file, signalling the completion of transactions and clearing any residual buffers for new packets. Figure 17 is a summary of events in the order of their occurrence during a sensing exercise.

#### 6.3.1. Technical Implementation

1. Bill of materials and system programming

The system was constructed from an array of cost-effective modules, chosen to meet the technological demands of the architectural design and operational expectations. A consideration of off-the-shelf modules was undertaken, which could allow for modifications of the circuit boards to suit the requirements of the system under development. This design allows for the easy replacement of any failed module without necessitating significant modifications to the system blueprint. Arduino IDE Version: 2.3.5 was utilised for software development due to its open-source nature and the availability of a wide community of support. Table 5 below shows the total cost of the monitoring system.

Some of the commercially available pesticide detection sensors are given in Table 6, shown below together with their respective prices.

2. Implementation consideration.

To ensure the smooth running of the system, suitable checks and balances were included in the development as follows:

- Error handling

GPRS disconnection

The MQTT function periodically checks the modem’s network connection status and attempts to reconnect in case of downtimes. This self-healing strategy guarantees the seamless publishing of messages on the Hive MQTT portal with minimal glitches.

SD write card failures

The SD card’s data write function ensures that the file to be written into is opened successfully. In case of a difficulty opening, an error log is posted on the serial monitor for debugging.

Bulk SMS

The function handling multicasting of the SMS to well users reports issues affecting its inability to successfully deliver messages by logging error messages for a diagnosis and correction.

- Scalability

Well expansion

The system design allows for the expansion of the network by allowing additional wells to be brought in for the monitoring exercise. The current configuration is designed for three wells; however, more wells can be accommodated by altering the handle received packet function to include more sources.

Use of alternative protocols

The modular structure programming employed in the development of the code permits the application of other alternative communication protocols like Zigbee and Bluetooth. Relevant libraries supporting the protocols need to be downloaded, and the setup () and loop () functions need to be adapted for the new protocol. Additionally, the MQTT function can support additional topics publishable in the portal.

Dynamic well identification

The well Id is determined dynamically using the comma index method. This code can handle strings with commas in different positions, such that if the format of the message changes, as long as there is a comma in the string, the code adapts and extracts the substring before the first comma.

- Power efficiency

There are elaborate schemes implemented to save power by introducing low-power modes of operation or sleep modes for modules to save power when not in active duty. During idle intervals, the system can dynamically slow down its operations, minimizing the active power drawn, like entering sleep modes only to wake up after a certain amount of time.

The sensor nodes are powered by an off-grid renewable energy resource consisting of a solar photovoltaic module (PV), a charge controller, and a battery. The solar panel is a Phaesun 20 W and provides a 1.14 A charging current during the day by converting solar irradiance to electrical energy. The energy storage element is a Zeus sealed lead acid (SLA) 12 V, 7 Ah battery. This battery is charged via a 12 V, 10 A pulse width modulation (PWM) charge controller, which in addition to managing the charging process, also offers protection against an overcharge, over discharge, short-circuit, and reverse polarity. This energy subsystem configuration ensures a continuous supply of power to sensor nodes, even in cloudy weather conditions. The power consumption was optimised through low-power coding schemes, resulting in an average current draw of 61 mA, yielding a battery backup duration of approximately 11 days without solar input.

#### 6.3.2. Estimated Power Consumption

Component

$$\begin{array}{l} \mathrm{Sensors}\;\mathrm{Estimated}\;\mathrm{current}\;\mathrm{during}\;\mathrm{LoRa}\;\mathrm{Tx}\;\mathrm{burst}\;=\;60\;\mathrm{mA}\\ \;\;\;\;\;\;\;\mathrm{Estimated}\;\mathrm{current}\;\mathrm{during}\;\mathrm{idle}\;\mathrm{state}\;=\;50\;\mathrm{mA}\\ \;\;\;\;\;\;\;\;\;\;\;\;\;\;\mathrm{Average}\;\mathrm{current}\;\mathrm{draw}=55\;\mathrm{mA} \end{array}$$

Charge controller

Self-consumption of charge controller = 6 mA                  Total average current drawn = 55 + 6 = 61 mA

Energy demand

$$\begin{array}{l} \;\;\;\;\;\mathrm{Battery}\;\mathrm{capacity}=12\;V\times 7\;Ah=84\;Wh\\ \;\;\;\;\;\mathrm{Operating}\;\mathrm{voltage}\;5\;V\\ \;\;\;\;\;\;\;\;\;\;\;\mathrm{Power}\;=\;V\times I=5\;V\times 0.061\;A=0.305\;W\\ \;\;\;\;\;\;\;\;\;\;\;\;\;\mathrm{Battery-only}\;\mathrm{run}\;\mathrm{time}\;=\;84\;Wh÷0.305\;W\\ \;\;\;\;\;\;\;\;\;\;\;\;\;\;\;\;\;\;\;\;\;\;=\;275\;h\sim 11.5\;days \end{array}$$

So, the sensor nodes can run for about 11 days without any solar input.

- Solar panel sizing

Solar panel peak power = 20 W;

Average sunshine hours in a day = 6 h/day;

Daily energy from panel ~20 W×6 h=120 Wh/day;

Charge controller conversion efficiency ~80%;

Usable energy ≈120×0.8=96 Wh/day (this is more than enough to replenish the daily energy (0.305×24=7.32 Wh) and charge the battery after depletion). 

The solar panel selected for this system delivers 1.14 A under standard operating conditions. The average daily sunlight hours of the deployment area from measurements are 6 h, leading to the generation of approximately 120 Wh of energy on a daily basis. Considering the losses from the charge controller and wiring, the usable energy per day is 96 Wh. This energy yield exceeds the daily energy requirement of a single sensor node, which is about 7.32 Wh. The surplus energy available, amounting to 88.68 Wh, serves two functions:

- This energy can fully recharge the battery in a day after many days of poor weather conditions.
- The battery can support the system in a working condition for 11 days without an external energy input.

The selection of this size ensures that more sensor nodes can be integrated in the system without additional changes to the hardware.

## 7. Conclusions

This study has demonstrated the implementation of a wireless network in monitoring the pesticide prevalence in shallow wells. To combat the menace of waterborne diseases, which have increasingly been a challenge to human health, the continuous monitoring of water sources is a significant step towards the realisation of that vision. Findings from this study establish that embracing user-friendly approaches enhances citizen involvement in volunteer monitoring activities and expands areas under investigation. The user interface system developed was simple to follow and had reduced interaction steps, enabled by exposing only two push buttons. Only motor skills were required to load data into the sensor nodes, avoiding a keyboard which could lead to possible typing errors.

IoT-based WSN systems support M2M communications, reducing logistical challenges of sample collections and further promoting data sharing and real-time updates. This approach facilitates widespread data access and improves the decision-making process. According to this research, modelling was instrumental in exposing challenges of the packet loss, which led to the addition of a feedback loop to improve data reliability. A notable feature of the system is its resilience against disruptive weather, achieved through an energy subsystem that maintains a backup duration of 11 days. The development of autonomous sensors specifically tailored for pesticide detection represents a significant step toward minimizing human involvement in the monitoring process. This advancement has the potential to usher in a new era in pesticide surveillance and environmental monitoring.

## Figures and Tables

**Table 1:** Water monitoring case studies.

Case Study	Technology/Approach	Results
Water quality monitoring in Krishna River (Karnataka, India) [62].	IoT-based water quality monitoring. Statistical analysis for collected data.	One-way ANOVA was more effective in analysis than two-way ANOVA. Variations in parameters affecting water quality influenced by season.
Water pollution monitoring in Tuhuando river, Ecuador [63].	IoT-based WSN system. Three node points based on population density and four data collection times per day. Application of quantitative metric of balance (QMB). Data analysing utilising supervised classification.	Data matrix reduced by 97% of original size. Achieved classification performance beyond 90%.
Monitoring of drinking water quality in Najaf, Iraq [64].	IoT-based WSN with Wi-Fi utilised for node–server communication. Data collected from 5 water stations. Programmable logic controller (PLC) used as the control unit.	Water quality parameters were below those prescribed by the World Health Organization.
Monitoring quality of drinking water sources in Gataia, Romania [65].	IoT-based system utilising Bluetooth radio frequency communication for acquisition of data. Five water sources were chosen and tested for three consecutive days.	Water from Tabor water pump source was not suitable for consumption. All the sources did not meet excellent quality of drinkable water.
Analysing a complex water quality dataset from Freiberger Mulde river in Saxony, Germany, to evaluate and optimise water quality variables and monitoring sites [66].	Using quantitative methods to measure information from a monitoring network and applying PCA results to support outcome. Evaluating trade-offs between information of monitoring network and expenses of monitoring activities. A total of 364 chosen measuring points, where 27 are on the mainstream and 337 are on the tributaries.	Main causes of variations in water quality highlighted as mining, weathering, seasons, and waste water discharge. Warm weather favoured greater variations in the factors affecting water quality. Monitoring more parameters at fewer sites proved less costly.
Water quality monitoring in Zhanghe River, China, by using (UAV) drone multispectral imagery and ML algorithms [67].	Acquiring high-resolution multispectral images using UAV flight missions. Collection of 45 samples from 5 sections of the river for ground-based lab testing of water parameters. Using ensemble method stacking ML to achieve better prediction results.	Non-linear models both using one input or multiple variables produced more accurate predictions than linear models except for chlorophyll-a, which performed particularly well despite being single-variable linear model. Estimating water quality parameters from remote sensing data shows a complex relationship between spectral information and parameters such as total nitrogen, total phosphors, and permanganate index.
Monitoring of water quality parameters using mixed online and portable methods in Umbulan drinking water processing outlet, Indonesia [68].	Using supervisory control and data acquisition to populate water quality parameters into an online server for 30 days. Measurement of water quality parameters using portable devices once a week for comparison with the automated system. Data processing and display using Scada human machine interface.	Offline readings and sensor values had negligible differences throughout the month. Average water quality parameters using online system indicated high stability.
Assessment of water quality based on satellite imagery data, multisensor cruise device, and deep neural network in Qingcaosha Reservoir, Shanghai [69].	Satellite images of the reservoir from sentinel-2 were obtained and processed. Water quality parameters for the day were captured using cruise BioFish along a designated route in the reservoir. Relationships of collected data were established using ML algorithms. Statistical performance metrics were used to evaluate the models’ accuracy.	Improved deep embedding clustering presented best performance in accuracy and stability over the other algorithms. Performance of each model increased with increase in size of training data.

**Table 2:** Labels for participants.

S.no	WELL A	WELL B	WELL C
1	P001A	P001B	P001C
2	P002A	P002B	P002C
3	P003A	P003B	P003C

**Table 3:** Performance analysis of models.

Model	Mean Absolute Error (MAE)	Root Mean Square Error (RMSE)	Standard Deviation (σ)
Okumura–Hata	29.7657	31.9179	7.6186
FSPL	42.4517	42.5075	2.2369
COST 231-Hata	6.1379	7.9247	7.6186
Log-distance	5.1611	6.2493	6.4187

**Table 4:** Participant demographics and data entry times during prototype sensor testing.

Participant Id	Trial1 (s)	Trial2 (s)	Trial3 (s)	Trial4 (s)	Trial5 (s)	Trial6 (s)	Age Set (yrs)	Education
V001	29.48	26.18	21.76	17.34	17.75	18.05	18–35	O’level
V002	30.01	25.65	23.10	19.00	19.00	18.28	18–35	Primary dropout
V003	28.66	25.00	20.59	17.53	18.11	17.53	18–35	Post sec
V004	29.89	27.04	21.37	18.08	17.21	17.06	36–60	Post sec
V005	30.56	26.79	22.67	18.00	17.67	17.15	36–60	O’level
V006	28.08	27.59	24.70	18.30	17.68	17.10	36–60	Primary dropout
V007	31.98	27.38	22.51	20.52	19.41	18.87	>60	Post sec
V008	31.72	26.11	22.05	19.00	18.63	17.12	>60	O’level
V009	30.11	28.14	22.49	21.00	19.57	18.95	>60	Primary dropout

**Table 5:** The cost of components utilised in the architecture.

Item	Cost in GBP
Sensor’s modules	
Arduino Uno R3 expansion board	22.26
PN532 NFC expansion board	32.22
LCD keypad shield 2 × 16	7.99
RFID tag 13.56 MHz	1.78
LoRa transceiver module—915 MHz	10.50
Glyphosate dipstick sensor	21.73
Simple Spring Antenna—915 MHz	0.80
Base station	
Arduino Uno R4 expansion board	21.45
Fona 808 shield GSM/GPS	40.28
LCD keypad shield 2 × 16	7.99
LoRa transceiver module—915 MHz	10.50
Simple Spring Antenna—915 MHz	0.80
SD card 2 GB class 6 SLC	27.06
SD card shield v4 board	12.55
Energy harvesting system	
12 V sealed lead acid (SLA), 7 Ah battery	25.45
R-78W DC/DC converter 5 V	7.73
12 V, 10 A Solar Charge Controller	37.13
Phaesun 20 W Photovoltaic Solar Panel	27.32
Total cost	315.54

**Table 6:** Cost of commercial pesticide detectors.

Item	Cost in GBP
Waters Acquity UPC2 system with PDA	23,734.00
Nanbei confocal Raman Microscope	33,220.00
Sciex 6500 + Triple Quad LC-MS/MS	229,629.63
Metrohm Misa SERS Raman	30,637.95
Agilent 6460C QQQ MS system with 1290 UHPLC front-end	110,760.00
Shimadzu prominence-i LC-2030C plus HPLC	19,779.00

## Data Availability

The raw data supporting the conclusions of this article will be made available by the authors on request.

## Appendix A. List of Analytes Tested Using Analytical Methods for Validation

2-Phénylphénol, Acequinocyl, Acétochlor, Acrinathrin, Alachlor, Aldrin, Allethrin, Allidochlor, alpha-BHC, alpha-Endosulfan, Anthraquinone, Benfluralin, beta-BHC, beta-Endosulfan, Bifenthrin, Biphenyl, Bromophos, Bromophos-ethyl, Bromopropylate, Cadusafos, Captafol, Captan, Carbophenothion, Chlorbenside, Chlorfenapyr, Chlorfenson, Chlorobenzilate, Chloroneb, Chlorothalonil, Chlorpropham, Chlorpyrifos-methyl, Chlorthal-dimethyl, Chlorthiophos-1, Chlorthiophos-2, Chlorthiophos-3, Chlozolinate, cis-Chlordane, cis-Nonachlor, Clomazone, Coumaphos, Cyfluthrin-2, Cyfluthrin-3, Cyfluthrin-4, Cypermethrin-1, Cypermethrin-2, Cypermethrin-3, Cypermethrin-4, Cyproconazole, Cyprodinil, delta-BHC, Deltamethrin-1 (Tralomethrin deg.-1), Deltamethrin-2 (Tralomethrin deg.-2), Dichlobenil, Dichlofluanid, Dichlorvos, Dichlorvos-d6 (ISTD), Dicloran, Dieldrin, Dimethachlor, Diphenamid, Diphenylamine, Disulfoton, Edifenphos, Endosulfansulfate, Endrin, EPN, Ethalfluralin, Ethion, Ethoprophos, Etridiazol, Fenamiphos, Fenchlorphos, Fenitrothion, Fenvalerate-1, Fenvalerate-2 (Esfenvalerate), Fipronil, Fluazifop-P-butyl, Flucythrinate-1, Flucythrinate-2, Fludioxonil, Fluquinconazole, Fluridone, Flusilazole, Flutolanil, Flutriafol, Folpet, Fonofos, gamma-BHC (Lindane), Glyphosate, Heptachlor, Heptachlor-exo-epoxide, Hexachlorobenzene, Hexazinone, Iprodione, Isazofos, lambda-Cyhalothrin, Leptophos, Metazachlor, Methacrifos, Methoxychlo, Mevinphos, Mirex, Nitralin, Nitrofen, Norflurazon, o,p′-DDD, p,p′-DDE, p,p′-DDT, Paclobutrazol, Parathion, Parathion-methyl, Penconazole, Pendimethalin, Permethrin-1, Permethrin-2, Phenothrin-1, Phenothrin-2, Phorate, Phosmet, Pretilachlor, Procymidone, Propachlor, Propargite, Propyzamide, Prothiofos, Pyraclofos, Pyrazophos, Pyridaphenthion, Pyriproxyfen, Quinalphos, Quintozene, Resmethrin-1, Resmethrin-2 (Bioresmethrin), Sulprofos, tau-Fluvalinate-1, tau-Fluvalinate-2, Tebuconazole, Tecnazene, Tefluthrin, Terbacil, Terbufos, Tetrachlorvinphos, Tetradifon, Tolclofos-methyl, Tolylfluanid, TPP (ISTD), trans-Chlordane, trans-Nonachlor, Triadimefon, Tricyclazole, Triflumizole, Trifluralin, and Vinclozolin.
